# A novel specimen shape for measurement of linear strain fields by means of digital image correlation

**DOI:** 10.1038/s41598-021-97085-x

**Published:** 2021-09-01

**Authors:** Nedaa Amraish, Andreas Reisinger, Dieter Pahr

**Affiliations:** 1 Institute for Lightweight Design and Structural Biomechanics, TU-Wien, Vienna, 1060 Austria; 2 Division Biomechanics, Department of Anatomy and Biomechanics, Karl Landsteiner University of Health Sciences, 3500 Krems a.D, Austria

**Keywords:** Materials science, Engineering, Biomedical engineering, Mechanical engineering

## Abstract

Strains on the surface of engineering structures or biological tissues are non-homogeneous. These strain fields can be captured by means of Digital Image Correlation (DIC). However, DIC strain field measurements are prone to noise and filtering of these fields influences measured strain gradients. This study aims to design a novel tensile test specimen showing two linear gradients, to measure full-field linear strain measurements on the surface of test specimens, and to investigate the accuracy of DIC strain measurements globally (full-field) and locally (strain gauges’ positions), with and without filtering of the DIC strain fields. Three materials were employed for this study: aluminium, polymer, and bovine bone. Normalized strain gradients were introduced that are load independent and evaluated at two local positions showing 3.6 and 6.9% strain change per mm. Such levels are typically found in human bones. At these two positions, two strain gauges were applied to check the experimental strain magnitudes. A third strain gauge was applied to measure the strain in a neutral position showing no gradient. The accuracy of the DIC field measurement was evaluated at two deformation stages (at $\approx$ 500 and 1750 μstrain) using the root mean square error (RMSE). The RMSE over the two linear strain fields was less than 500 μstrain for both deformation stages and all materials. Gaussian low-pass filter (LPF) reduced the DIC noise between 25% and 64% on average. As well, filtering improved the accuracy of the local normalized strain gradients measurements with relative difference less than 20% and 12% for the high- and low-gradient, respectively. In summary, a novel specimen shape and methodological approach are presented which are useful for evaluating and improving the accuracy of the DIC measurement where non-homogeneous strain fields are expected such as on bone tissue due to their hierarchical structure.

## Introduction

Digital Image Correlation (DIC) measures full-field strain on the surface of specimens by capturing images during mechanical testing. Due to its main advantage in measuring full-field surface strains on specimens with irregular shape and different sizes, two- and three-dimensional (2D and 3D) DIC systems have been used to characterize a wide range of materials under different experimental setups^1–4^. Despite the advantages of using DIC systems to capture full-field strain distributions, noise in DIC strain measurements is non-negligible^5–9^. DIC measuring parameters, like facet and step sizes, can be optimized to reduce the noise of the full-field data. However, in case of measuring on a surface where non-homogeneous strain fields are expected, the typical optimized DIC parameters might have a counter effect because the risk of losing information is higher, specially at locations of higher strain gradients^10–12^. Additionally, different filtering techniques can be applied to reduce the noise in DIC measurements, which, if not carefully applied, can have an effect of over smoothing leading to loss in information^8,13^, filtering can be powerful when the true strain distribution is known a priori.

For homogeneous strain fields, such as fields expected on regular aluminium^8,14,15^ or steel^16,17^ specimens with constant cross-section, DIC measurements can be verified using strain gauges (SGs) or extensometers. However, for non-homogeneous strain fields, such as strains found on complex structures^11,18–22^ or biological tissues like, the human femur^9,23,24^, bone from different animal models^25–28^, the vertebrae^29–33^, or soft tissues^34,35^, verifying the full-field DIC strain measurements can become challenging.

Various studies examined the accuracy of DIC strain measurements and showed that the strain on the measured surfaces can be overestimated^9,15,23,27^. The precision of DIC measurements is acceptable when specimens are deforming in the linear-elastic region, but after yielding the standard deviation increases vastly^36,37^. Two options could be employed for investigating the accuracy of DIC strain measurements, either by verifying the measurement at the position of measurement device^24,27,38–40^, or by means of finite element (FE) models for the full-field strain distribution^8,9,12^. Despite the high accuracy of SGs and extensometers, their measurement is limited to a single point, and can be compared to DIC measurements only by averaging the full-field data over the SG area^15,39,41^, which is only advantageous for homogeneous strain fields. In contrast, FE models can give accurate prediction of the full-field strain measurements, but it is important to know the geometry, material behaviour and boundary conditions. With knowledge of these parameters FE models are a suitable way to evaluate strain non-homogeneity^12,42,43^. For example, Liu et al.^12^, investigated experimentally the strain concentration on hydrogel specimens in the presence of large strain gradients, they found a very good agreement between the FE results and the measured DIC full-field strain, however, 2D DIC was employed which is less useful for objects with curvatures, like the human femur.

Non-homogeneous strain fields were measured on the surface of biological tissues^24,27,44,45^. For example, on the neck of the human femur, the normalized strain gradient changes by about 7% per mm on average (more in the next section). DIC measurements is advantageous not only for measuring non-homogeneous strain fields, but also for measuring strain gradients^46–48^. Various studies reported DIC full-field strains without reporting the accuracy of the measurement or validating it against another method, Tsirigotis et al.^49^ found that the surface strain, on bovine cancellous bone under compression load, showed steep strain gradients. Likewise, Palanca et al.^50^ and Grassi et al.^51^ measured full-field surface strain on the superior neck of human femurs. However, the strain results were not validated against another measurement method, and Tsirigotis et al.^49^ used 2D DIC. Many DIC strain measurements on different bone models were verified using SGs only, and were used to verify FE models^22,27,38,52,53^. Despite the numerous studies on the accuracy of DIC measurements, only one study, Baldoni et al^8^, verified linear experimental full-field DIC strain measurement, the verification was against the theoretical solution and not against another measurement technique, and the strain gradient was not analyzed.

Other studies focused on testing the accuracy of DIC numerically. For instance, Wang et al.^11^, tested the accuracy of 2D DIC for non-homogeneous strains of numerically deformed images, they found that the uncertainty of DIC increases around the strain concentration regions,here the reference images were also captured by one camera only (2D DIC), and numerically deforming the images excludes the errors originated from the experiment’s environment. A verification of 3D DIC measurements for linear strain fields and strain gradients experimentally is still missing. To verify non-homogeneous strain fields measured by DIC, the strain gradients on the specimen’s surface must be known. Non-homogeneous strain fields such as linear or quadratic strain fields are good candidates since the analytical (theoretical) solution can be calculated beforehand. This study is focusing only on linear strain fields.

The objective of this study is to design a novel specimen shape where a well-defined linear gradient field can be measured and to investigate the accuracy of DIC full-field strain measurement globally and locally. This is the first study to systematically evaluate and validate non-homogeneous DIC strain measurements on surfaces where strain gradients are expected on biological and engineering materials. Measurements are done with a 3D DIC system and the noise in the DIC strain fields is reduced by applying Gauss low-pass filtering with optimal cutoff frequency. SGs are used to measure the experimental strain at pre-defined positions, which was compared to the DIC strain measurement locally. Summarizing, this work aims at verifying whether DIC measurements can capture gradient fields and local normalized strain gradients at specific positions in case of bone and for comparison purposes on typical engineering materials (aluminium and polymer).

## Materials and methods

### Normalized strain gradient

To explore the capability of a DIC system to measure linear strain fields on the surface of the test specimens, it is necessary to design a specimen shape where the strain on the surface of the specimen changes linearly during deformation. Different options are available to design such a specimen, either by changing the width or the thickness of the test specimen. In this study, it is intend to measure strain gradient magnitudes similar to that found on a surface of a proximal human femur. For this purpose, a load and size independent measured value - a normalized strain gradient - is defined. This is based on a normalized strain $\varepsilon _{\text {norm}}$ which is the difference between the strain at two points divided by their average quantity [Eq. (1)]. The gradient is obtained by dividing this normalized strain by the distance of these two points (Eq. (2)).

1 $$\begin{aligned}&\varepsilon _{\text {norm}}= \frac{|\varepsilon _{\mathbf {p1}} - \varepsilon _{\mathbf {p2}}|}{\frac{\varepsilon _{{\mathbf {p1}}} + \varepsilon _{{\mathbf {p2}}}}{2}} \end{aligned}$$

2 $$\begin{aligned}&\varepsilon _{\text {norm grad}}= \frac{\varepsilon _{\text {norm}}}{d} \left[ \frac{1}{\text {mm}}\right] \end{aligned}$$

where $\varepsilon _{\mathbf {p1}}$ and $\varepsilon _{\mathbf {p2}}$ in this work are equivalent strains $\varepsilon _{eq}$ which are measured at two locations ${\mathbf {p1}}$ and $\mathbf {p2}$ on the femur, and *d* is the distance between the two points (see in Appendix A, Supplementary Fig. 1 shows a SED map of a proximal femoral under physiological load^54^). The equivalent strain $\varepsilon _{eq}$ is obtained from the local strain energy density (SED) and elastic modulus (10 GPa) as follows:

3 $$\begin{aligned} \varepsilon _\text {{eq}}= \sqrt{\frac{2 SED}{E}} \end{aligned}$$

For the human femur, the normalized strain gradient is about 3.5 and 7.2% per mm on average in the head and neck regions, respectively (see Appendix A, Supplementary Table 1). This work aims to design a specimen shape which under deformation gives a linear strain field, and where two normalized strain gradients of around 3.5 and 7% per mm (regardless of the load applied) can be found on the surface.

### Analytical strain and specimen shape

A linear field in a tensile specimen can be generated by a specific specimen shape. In the following derivations we calculate the specimen shape. The exact strain distributions for comparison with DIC are determined by means of FE.

**Figure 1:**
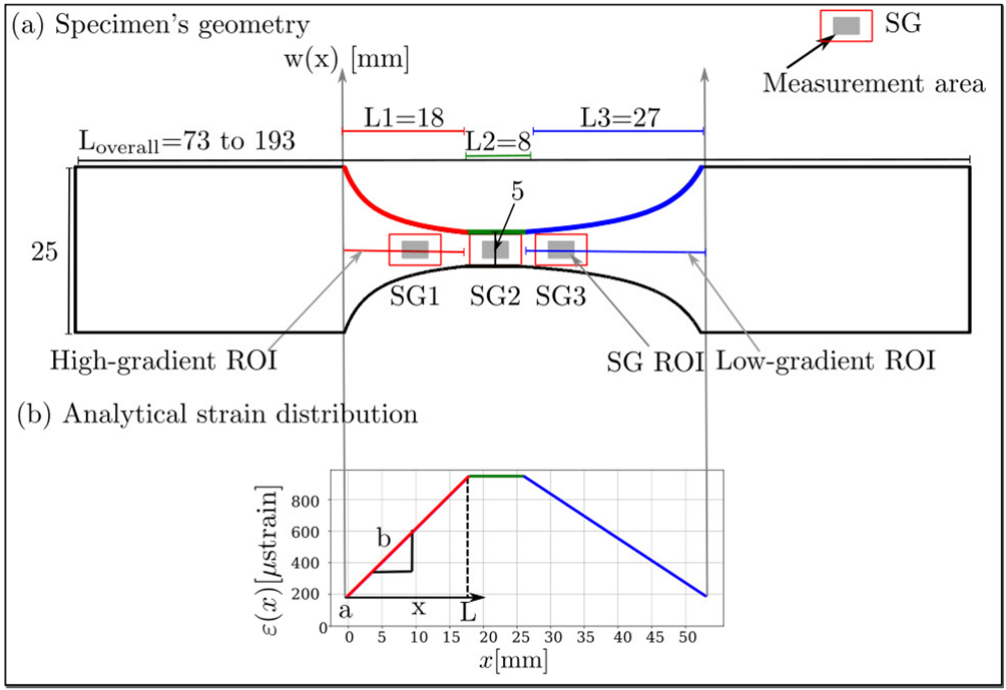
(**a**) Specimen’s geometry derived from Eq. (12), *w*(*x*) shows how the curvature of the specimen changes; in red and blue the high- and low-gradient ROIs are shown, respectively. L1 is the high-gradient ROI and where SG1 is placed. L2 is a neutral ROI connecting the high-gradient ROI with the low-gradient ROI, L2 shows constant strain and where SG2 is placed. L3 is the low-gradient ROI and where SG3 is placed. (**b**) The theoretical strain distribution along the specimen’s length. The high- and low-strain gradients are shown in red (steeper curve) and blue, respectively. Constant strain (in green) connects both gradient regions.

In the following uniaxiality is assumed, *w*(*x*) is the width (shape) of the specimen, and $\varepsilon (x)$ is the strain along *x*, see Fig. 1a and b. A one-dimensional linear strain field can be written as:

4 $$\begin{aligned} \varepsilon (x)= a + bx \end{aligned}$$

where *a* is a point on the specimen where the strain shows the far field value, *b* is the slope of the linear strain and *x* is a position along the specimen’s axis, see Fig. 1. Solving for *a* and *b* at the positions 0 and *L* gives:

5 $$\begin{aligned}&\text {at}\,\textit{x} = 0: \qquad \varepsilon (0)= a \end{aligned}$$

6 $$\begin{aligned}&\text {at}\, \textit{x} = \textit{L}: \qquad \varepsilon (L)= \varepsilon (0)+b \cdot L =\varepsilon (0)\cdot k \end{aligned}$$

where the maximum strain $\varepsilon (L)$ can be linked to the far field strain $\varepsilon (0)$ by a concentration factor *k*. Solving for the slope of the linear equation, *b*:

7 $$\begin{aligned} b= \frac{\varepsilon (0)}{L}\cdot (k-1) \end{aligned}$$

The strain at any point between *a* and *L* can be calculated via inserting *a* and *b* in Eq. (4):

8 $$\begin{aligned} \varepsilon (x)= \varepsilon (0) + \frac{\varepsilon (0)}{L}\cdot (k-1) \cdot x = \varepsilon (0) \Big (1+ (k-1) \cdot \frac{x}{L}\Big ) \end{aligned}$$

Due to the asymmetric specimen shape as shown in Fig. 1, two different strain gradients can be realized. For example, if $\varepsilon (0)$, *L* and *k* are prescribed, the strain function and the normalized strain gradient [Eq. (2)] along the specimen’s length can be computed. The normalized strain gradient changes between 4.4 and 22% per mm, and between 3 and 14% per mm for specimen’s length of 18 and 27 mm, respectively. Two positions were selected relatively close to the middle of the specimen for the normalized strain gradients investigation. These are the nearest values to the two normalized strain gradients found on the surface of the human femur (3.5 and 7.3% per mm), which are 3.6 and 6.9% per mm for a specimen’s length of 18 and 27 mm, respectively.

The unknown width of the specimen *w*(*x*) follows from the equilibrium i.e. the force experienced along the specimen’s length is constant at one loading stage:

9 $$\begin{aligned} F(0)= F(x) \end{aligned}$$

Therefore, the equation can be rewritten in terms of stress ($\sigma =E \cdot \varepsilon$) and cross-sectional area ($A=2w(0) \cdot t$):

10 $$\begin{aligned} E \cdot \varepsilon (0) \cdot 2w(0) \cdot t= E \cdot \varepsilon (x) \cdot 2w(x) \cdot t \end{aligned}$$

where *t* is the thickness of the specimen (which is constant), *w*(0) is the half-width of the specimen and equals 12.5 mm, see Fig. 1a. Equation (10) can be rewritten as:

11 $$\begin{aligned} w(x) = \frac{\varepsilon (0) \cdot w(0)}{\varepsilon (x)} \end{aligned}$$

Solving for *w*(*x*):

12 $$\begin{aligned} w(x) = \frac{\varepsilon (0) \cdot w(0)}{\varepsilon (0) \Big (\frac{ L+(k-1) \cdot x}{L}\Big )} = \frac{w(0)}{1+(k-1) \cdot \frac{x}{L}} \end{aligned}$$

Figure 1a shows the specimen’s geometry for *k* = 5 by plotting *w*(*x*) from Eq. (12) for two lengths, in red and blue in Fig. 1a. The size limitation in this work was on the one hand the specimen width of 25 mm for bone and on the other hand the specimen width of 5 mm in SG2 ROI for the application of SG2. The overall length of the specimen was 193 mm for aluminium and polymer specimens and was 73 mm for bovine bone specimens. The length of the Region of Interest (ROI) is 53 mm and is divided into three regions (L1, L2 and L3). L1 is 18 mm long (high-gradient ROI, showing 6.9% per mm normalized strain gradient), L2 is 8 mm long (constant strain, no gradient), and L3 is 27 mm long (low-gradient ROI, showing 3.6% per mm normalized strain gradient). In these three regions, three SGs are applied at specific locations. Three ROIs are investigated in this study: the high- and low-gradient ROIs and the SGs ROIs. Figure 1b shows the linear strain (in red and blue) along the shape of the specimen.

### SGs strain

Strains were recorded with one-element, 120-ohm rectangular strain gauge (K-CLY4-0030-1-120-3-020, HBM, Darmstadt, Germany). The strain in the loading direction was recorded (acquisition frequency 5 Hz) using a QuantumX data acquisition (HBM, Darmstadt, Germany).

The positions of the three SGs are depicted in Fig. 1. SG2 was applied at L2 where the strain is constant. This strain was used to calibrate the FE models as described below for the FE model. SG1 and SG3 were applied where the normalized strain gradient is 6.9 and 3.6% per mm, which as explained above can be found on the surface of the femoral neck and head, respectively.

### Numerical strain (FE model)

**Figure 2:**
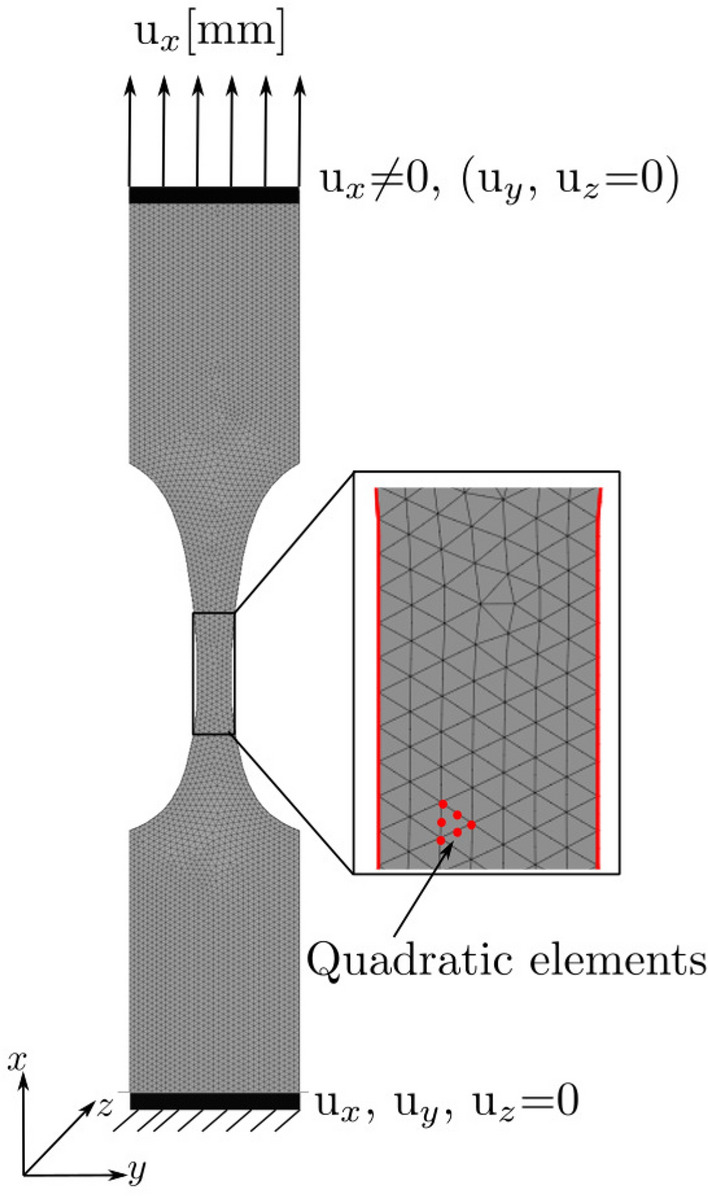
FE model including boundary conditions and mesh. The displacement was applied on the top of the specimen, while the bottom was constrained to mimic the experimental conditions. No material parameters (Elastic modulus) were assigned to the specimens because only strains were calculated and the FE simulation is displacement control. A poisson’s ratio of 0.3 was assigned for all specimens.

The analytical strain field from Eq. (8) does not consider the Poisson effect. To check the analytical model and verify the accuracy of the DIC measured strains, FE models were generated to provide accurate strains. The FE models were created for each specimen using an open source Calculix solver (PrePoMax, v0.6.0). The specimens’ geometry are as shown in Fig. 1a. The specimens were meshed with tetrahedral (C3D10) second-order elements of the size 0.8 mm, see Fig. 2. To scale the FE strain to the strain measured by SG2, 1 mm displacement ($u_x$) was imposed on the specimen simulating a uniaxial tensile test in the x-direction. The obtained FE strain was scaled so that the FE strain is equal to the strain in SG2. This is possible because of the linear elastic system.

### DIC strain measurements

DIC computes full-field strain on the surface of the specimen. More information is available here^17^ on how DIC computes strain. For this study, the surface strain was computed with a facet (subset) size of $19\times 19$ pixels and a facet step of 16 pixels (50% overlapping), these parameters are recommended by the manufacturer for 6 Megapixel CCD cameras. The strain in the loading direction were exported from the ARAMIS Professional software (v6.3.1; GOM GmbH, Germany) along the node number (> 600 measurement points) and x-, y-, z-coordinates, for plotting and post-processing with Python SciPy.

### Specimen preparation

The specimen shape was produced in aluminium (ALMG3 (AW-5754)) (*n* = 5), polymer (Polyacetal POM-Copolymer) (*n* = 5) and Bovine bone (*n* = 5). Aluminium and polymer specimens were manufactured by means of a numerical controlled machine (CNC Router BZT PFX 700, BZT Maschinenbau GmbH, Leopoldshöhe, Germany) from aluminium and polymer plates of 1.5 mm and 4 mm thickness, respectively.

**Figure 3:**
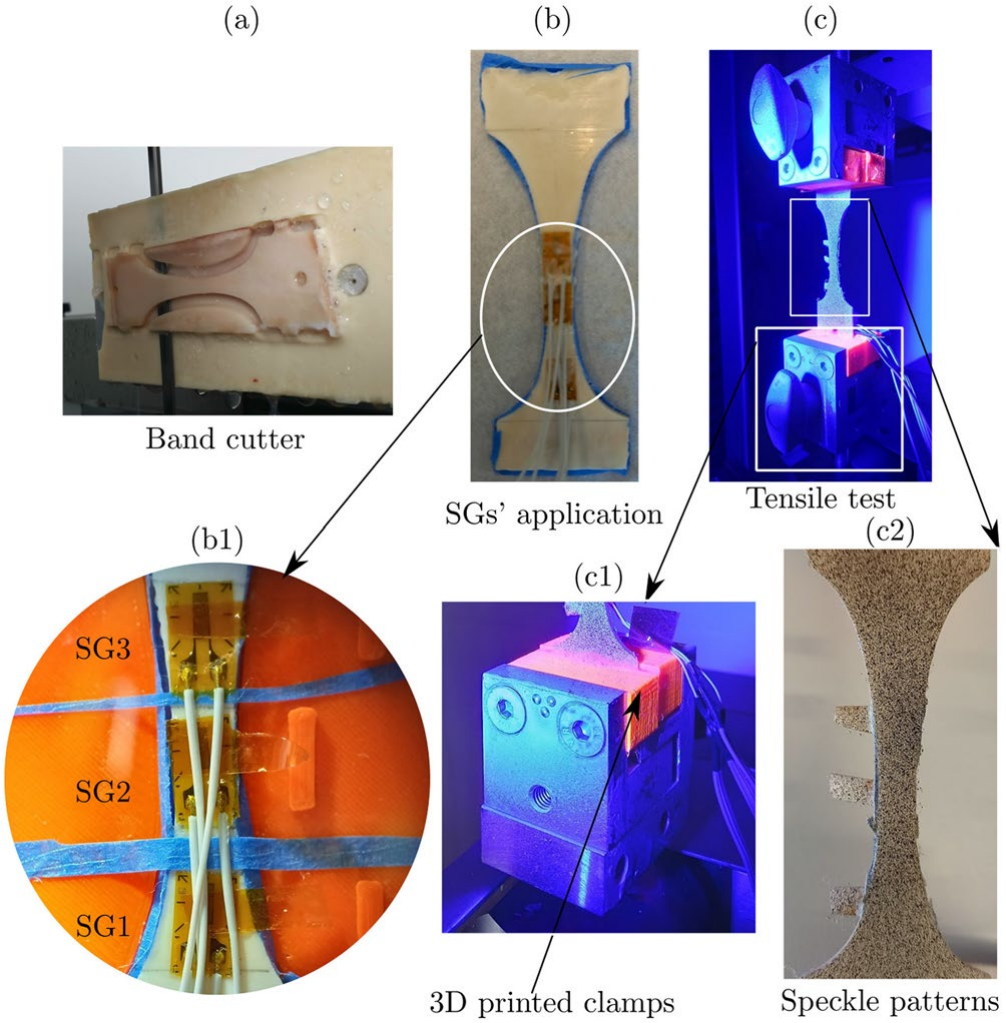
Preparation steps of the bovine bone specimens. (**a**) Bone specimens were sliced using an Exakt cutter, (**b**) SG1, SG2 and SG3 were applied on the specimen’s back, (**c**) mechanical test setup, (**c1**) the bone specimen was fixed to external clamps, and (**c2**) speckle patterns were applied on the specimen’s front.

For bovine bone, two fresh compact femurs of bovines (18–24 months old) were obtained from the local butcher. The mid-diaphysis of the femur was cut, the hollow cylinder of the femur shaft was then cut into rough rectangular beams using a hand saw. After that, the beams were embedded into epoxy mould which was then fixed into a CNC machine to obtain the shape of the specimen. Finally, using a slice cutting machine (Exakt 300 CL Band System, EXAKT Advanced Technologies GmbH, Norderstedt, Germany) longitudinal specimens were sliced (thickness of 2 mm), see Fig. 3a. During the whole preparation steps and until testing, the bone specimens were kept wet with phosphate buffered saline (PBS) solution and when not used, the specimens were preserved in a − 20 °C freezer.

Three SGs were applied on one face of the test specimen as in Fig. 3b while the other face was covered with paper tape to protect it against any glue resins during the SGs’ application. First, using a light microscope, the position of the SG was precisely marked on the surface of the specimen. Second, two component glue (Methyl methacrylate, HBM, Darmstadt, Germany) were mixed and applied on the bone surface and then each SG was carefully applied. Each specimen was then fixed to external clamps by gluing the specimen to 3D printed parts using 5 mins two components epoxy (Fiber-reinforced composite, Waldenbuch, Germany) as in Fig. 3c1. Speckle patterns were applied to the other surface using a high precision airbrush (Profi-AirBrush, Germany), as shown in Fig. 3c2. The airbrush settings were adjusted (air pressure of 150 kPa, 3 turns of the airbrush opening, and 9 cm distance between the airbrush and the specimen) to obtain a speckle size of 3−5 pixels with a random distribution (coverage 45–50%)^5^. Finally, the specimen was clamped to the tensile testing machine and was exposed to the blue light for the DIC system see Fig. 3c. Similar procedure was followed for the aluminium and polymer specimens, only another glue (Cyanoacrylate, HBM, Darmstadt, Germany) was applied to fix the SGs on the specimens’ surface as recommended by the manufacturer of the SGs.

### Mechanical tests

The test specimens were mounted on a Zwick (Z030) machine (ZwickRoell GmbH, Germany) with force cell up to 30 kN. 3D DIC system (ARAMIS 150/6M/Rev.02,GOM, Braunschweig, Germany) was set up with two CCD cameras. The cameras were positioned perpendicular to the specimen at 35 cm distance. The SGs cables were welded to an adaptor (full-bridge) which was connected to a QuantumX DAQ device (HBM, Darmstadt, Germany) for data acquisition. Finally, the specimens were subjected to a uniaxial tensile load along the vertical direction (displacement control) with cross-head movement of 0.5 mm/min till fracture. The acquisition rate was synchronized between the SG’s and the DIC system at 5 Hz.

### Data evaluation

**Figure 4:**
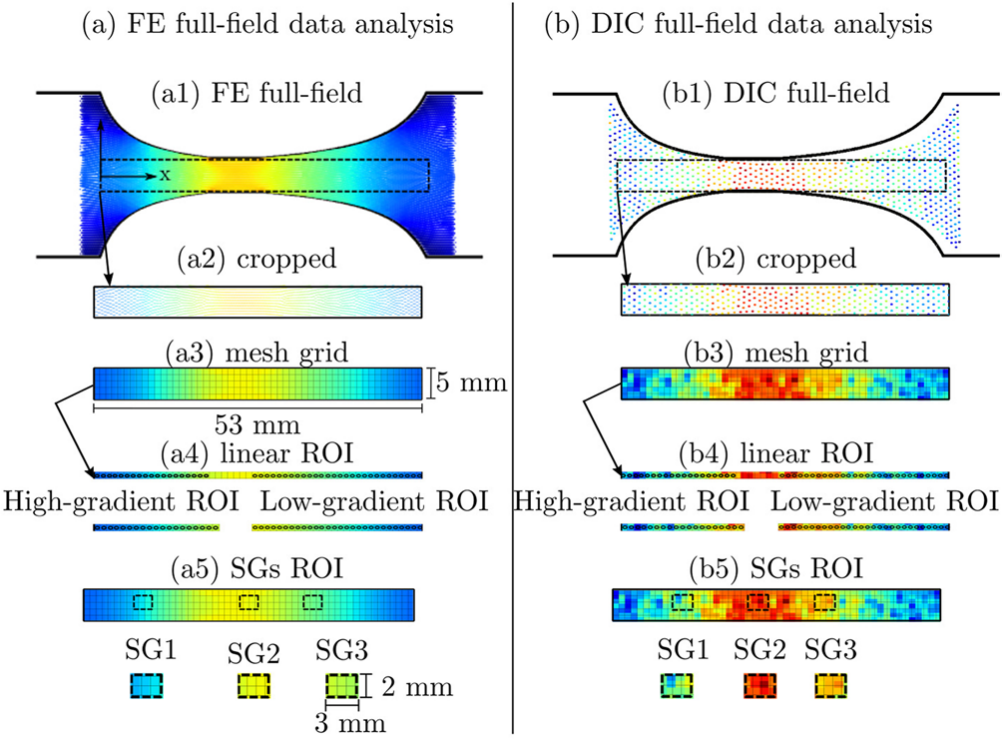
RMSE computational method. (**a**, **b**) FE and DIC full-field data analysis respectively, (**a1**, **b1**) the full-field surface strain from FE and DIC, (**a2**, **b2**) cropping of the linear regions excluding the boundary nodes, (**a3**, **b3**) a 53 mm × 5 mm mesh grid for aligning the measurement points into a regular grid, (**a4**, **b4**) the high- and low-gradient ROIs from the middle line along the specimen’s length were extracted, (**a5**, **b5**) SGs ROIs were cropped at SG1 and SG3.

In this study, the strain measurements were defined over three ROIs: high- and low-gradient ROI, and SGs ROIs, see Fig. 4. The scaled full-field strain obtained from the FE model is considered as the reference strain. At the high- and low-gradient ROIs, the accuracy of the DIC strain measurements and the analytical strain computation are evaluated by means of the root mean square error (RMSE). At the SGs ROIs, the two experimental strains form DIC and the SGs are compared statistically, and the normalized strain gradients (6.9 and 3.6% per mm) are computed and compared to the reference obtained from FE.

Figure 4 shows the computational method of the RMSE:

- The full-field strain (Fig. 4a1, b1) obtained from both FE and DIC, respectively. They were cropped horizontally and vertically based on the x- and y- coordinates.
- The cropped fields were interpolated into a regular mesh grid of (53 mm $\times$ 5 mm) points, see Fig. 4a3, b3) to be able to compare values.
- The middle line of the interpolated fields was exported into a 1D matrix, see Fig. 4a4 and b4 for line plots along the centre of the specimen.
- The positions of the three SGs were cropped from the (53 mm $\times$ 5 mm) mesh grid for SG size of 3 mm $\times$ 2 mm, see Fig. 4a5 and b5 to compare values inside the SG region.
- The RMSE was calculated for the high- and low-gradient ROIs.

The RMSE equation is:

13 $$\begin{aligned} \text {RMSE}_{\text {Method}} = \sqrt{\frac{1}{n}\Sigma _{i=1}^{n}{\Big (\mathrm {^{FE}}\varepsilon _{i} - \mathrm {^{Method}}\varepsilon _{i}\Big )^2}} \end{aligned}$$

where $\hbox {RMSE}_{\text {Method}}$ refers to the RMSE computed for each method, i.e. $\hbox {RMSE}_{\text {DIC}}$ is the RMSE of the DIC strain measurements compared to FE strains, *n* is the number of strain measurement points (*i*) in a ROI, $\mathrm {^{FE}}\varepsilon _{i}$ is the value of the reference strain (FE), $\varepsilon _{i}$ is the value of the analytical or DIC strains.

The RMSE was evaluated at two deformation stages, hereinafter referred as ($\hbox {Stage}_{1}$ and $\hbox {Stage}_{2}$). $\hbox {Stage}_{1}$ is at approximately 500 μstrain, a low strain level similar to the noise level of the DIC measurement. $\hbox {Stage}_{2}$ is at approximately 1750 μstrain level which is found during normal ambulation and is comparable for bone deformation under physiological load^55^. Because the exact values of 500 and 1750 μstrain were not found in all the measurements of SG2 for all the test specimens, the nearest measurement points to 500 or 1750 μstrain were selected which were about 496 and 1698 μstrain, respectively. The nearest point had a difference of less than 3% to 500 or 1750 μstrain.

To answer the question of how well the local strain gradient can be captured with DIC compared to FE, the two normalized strain gradients (6.9 and 3.6% per mm) are computed as in Eq. (2). Basically, along the specimen’s axis, first the normalized strain [Eq. (1)] is computed by dividing the difference in strain measurement between two measurement points by their average quantity. Then, the normalized strain gradient is computed by dividing the normalized strain by the distance between the two measurement points, 2 mm.

### Noise reduction

Gaussian low-pass filter (LPF) with cutoff frequency of 2.5, which was found as the optimal cutoff frequency in our previous study^17^, was applied on the DIC full-field strain measurements to reduce the noise in the DIC strain measurements. The same cutoff frequency was applied on all stages independent of the load applied.

## Results

### Mechanical testing

**Figure 5:**
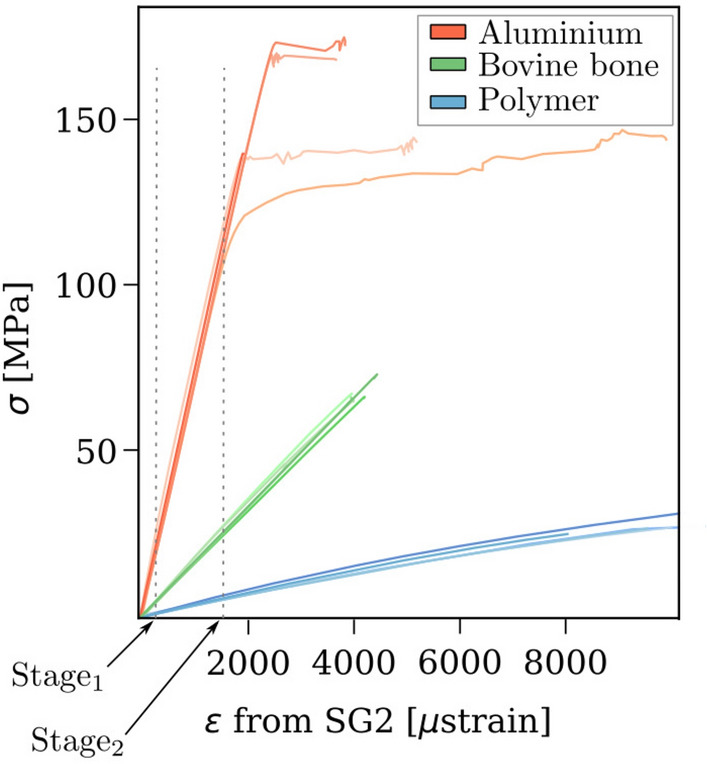
Stress–strain curve of all tested specimens. Aluminium in red, bovine bone in green and polymer in blue. The error evaluation was done at two deformation stages, approximately at 500 μstrain and 1750 μstrain. Five specimens were tested from each material.

The stress–strain curves of all tested specimens (five of each material), with strain obtained from SG2 (constant strain) are shown in Fig. 5. The two vertical dashed lines show the deformation stages ($\hbox {Stage}_{1}$ and $\hbox {Stage}_{2}$) at which the results were evaluated.

From the stress–strain curve, the elastic modulus was computed for the tested materials and it was on average 71.37 ± 2.03, 3.24 ± 0.25, and 16.92 ± 0.61 GPa for aluminum, polymer, and bovine bone, respectively (for more details, see Appendix B, Supplementary Table 2 lists the elastic modulus for all specimens. For bovine bone, results of the elastic modulus are in agreement with values found in the literature for cortical bone^56–58^).

### RMSE for the high- and low-gradient ROIs

**Figure 6:**
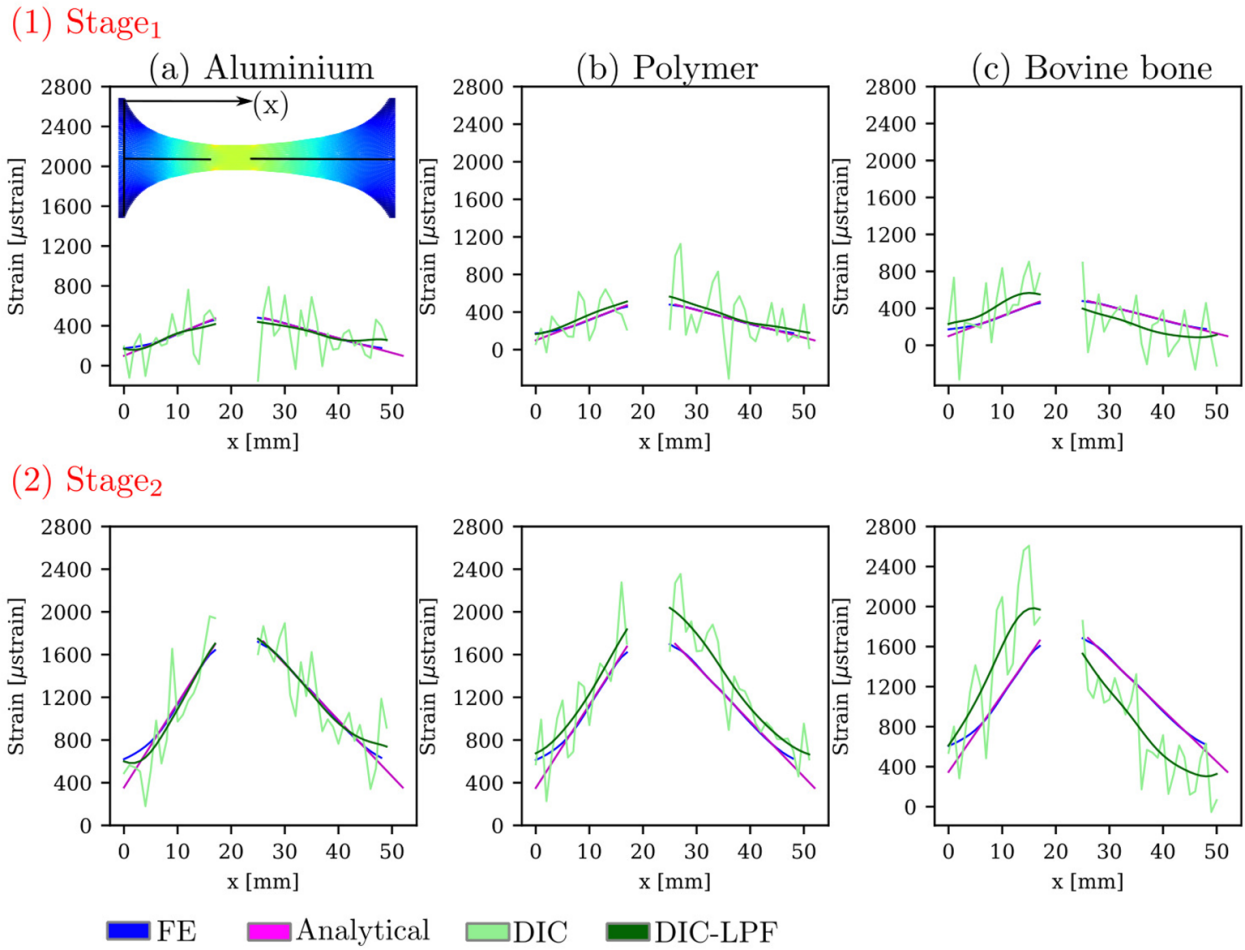
Full-Field linear strains at the two deformation stages, for (**a**) aluminium, (**b**) polymer, and (**c**) bovine bone. The strain is plotted along the specimen’s ROI (53 mm), the strain was obtained from the high- and low-gradient ROIs, as in Fig. 5a4 and b4. The FE, analytical, DIC and DIC filtered strain are plotted in blue, magenta, light-green and dark green, respectively. DIC-LPF refers to the DIC strain fields after Gaussian LPF was applied.

The RMSE was evaluated for the high- and low-gradient ROIs, as shown in Fig. 4c1 and c2. Figure 6 shows the two ROIs plotted along the specimen’s length at two deformation stages ($\hbox {Stage}_{1}$ and $\hbox {Stage}_{2}$). For aluminium and polymer, there is a good agreement between the FE strain, the analytical strain and the filtered DIC strain. For bovine bone, the DIC strain overestimated the strain in the high-gradient ROI at both deformation stages, in contrast to the low-gradient ROI where DIC strain underestimated the strain in comparison to FE strain. In all curves, the LPF successfully reduced the noise (the fluctuations) in the DIC strain measurements.

**Figure 7:**
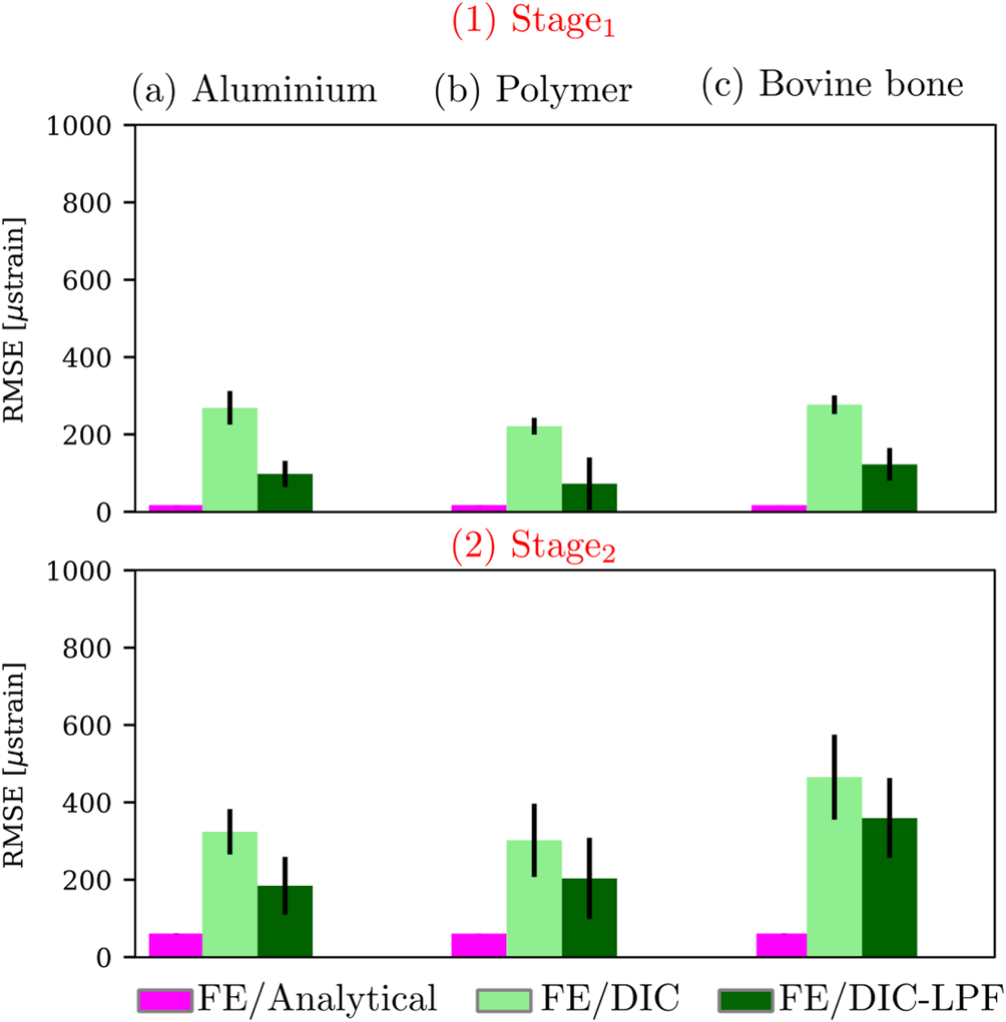
RMSE for the linear strain gradients ROIs at the two deformation stages for the three tested materials.

Figure 7 depicts the RMSE for the linear strain gradients ROIs at $\hbox {Stage}_{1}$ and $\hbox {Stage}_{2}$ for the three tested materials. The $\hbox {RMSE}_{\text {analytical}}$ deviated by less than 60 μstrain from the reference FE strain for all the tested materials. The $\hbox {RMSE}_{\text {DIC}}$ was about 400 μstrain when compared with the FE strain. Filtering of the DIC fields had a positive effect on the RMSE where it was reduced on average by 63% at $\hbox {Stage}_{1}$ and by 34% at $\hbox {Stage}_{2}$.

**Figure 8:**
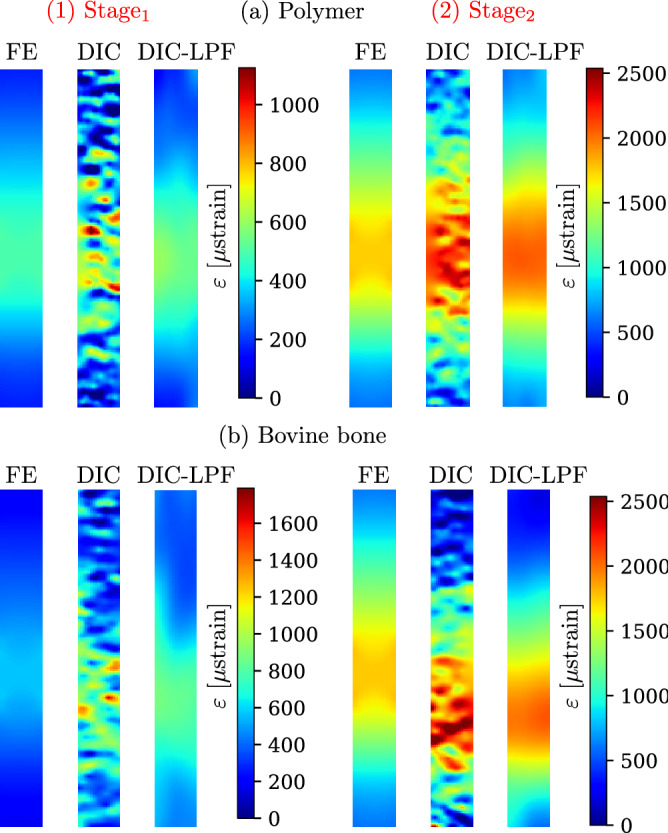
Two-dimensional visualization of the DIC full-field strain measurements of one polymer and one bovine bone specimens. At both deformation stages; the reference strain from the FE model for this specific specimen, the DIC strains (original and filtered) are shown.

Two-dimensional visualization of the full-field strains from FE and DIC are depicted in Fig. 8 for (a) one polymer and (b) one bovine bone specimens (The results of all the tested specimens are shown in Appendix C, Supplementary Fig. 2). Linear changes in the strain field cannot be recognized at $\hbox {Stage}_{1}$. On the contrary, at $\hbox {Stage}_{2}$, the linear strain field can be recognized, but corrupted with noise, which was then reduced when Gaussian LPF was applied. It is worth noting that the DIC strain fields shown in Fig. 8 are the raw data from the ARAMIS software without the application of any filtering, neither when the surface component was created, nor when the strain was calculated. The DIC-LPF fields are the DIC fields after the application of the Gaussian LPF using an in-house algorithm.

### Normalized strain gradient at SGs ROIs

**Figure 9:**
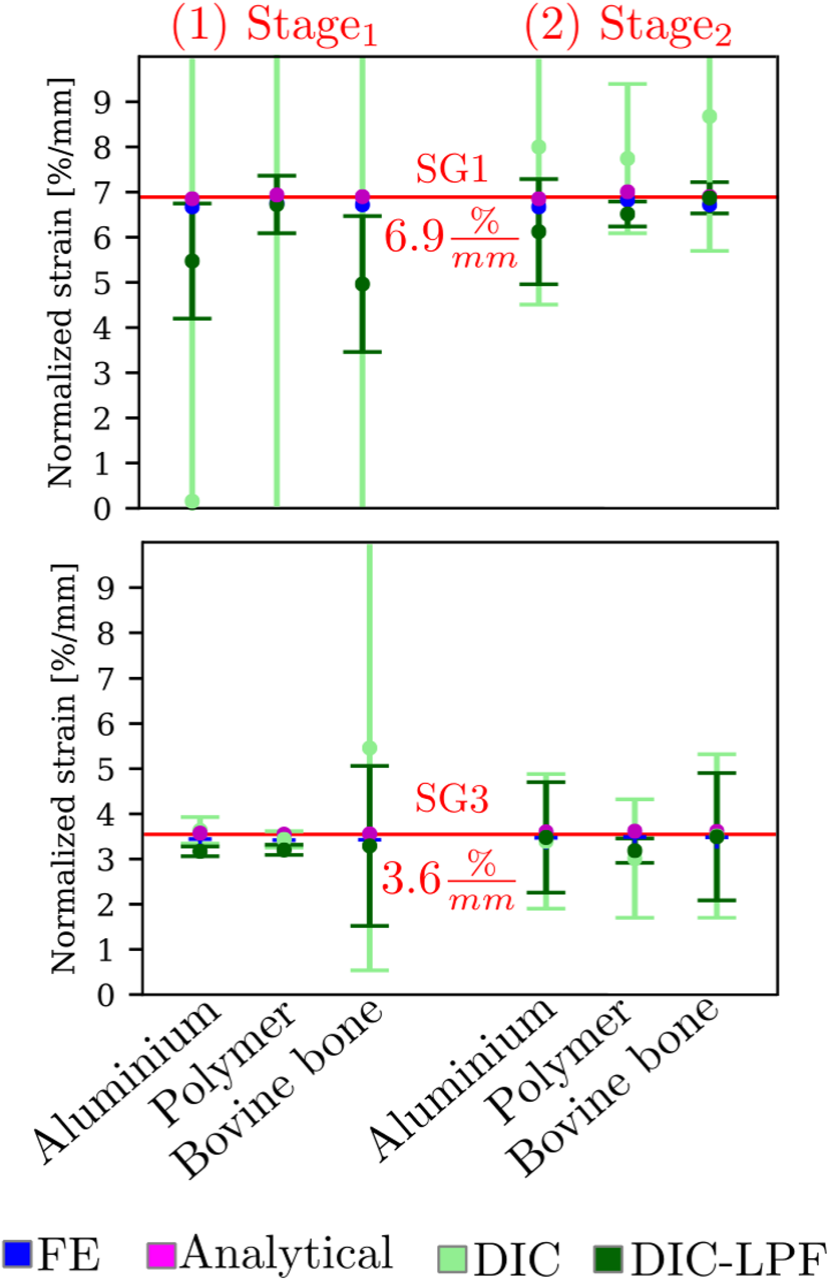
The normalized strain gradients at SG1 (6.9% per mm) and SG3 (3.6% per mm) is plotted for two deformation stages for aluminium, polymer, and bovine bone. The normalized strain gradient was calculated according to Eq. (2).

At the positions of the SGs, the corresponding FE and DIC strains were cropped as shown in Fig. 4a5 and b5. Figure 9 shows the normalized strain gradient at the positions of SG1 (6.9% per mm) and SG3 (3.6% per mm).

At SG1 (high-gradient), the DIC normalized strain gradient fails severely (with maximum difference to 6.9% per mm exceeding 90%) at $\hbox {Stage}_{1}$ and fails moderately (with maximum difference to the reference of 25%) at $\hbox {Stage}_{2}$. After applying the Gaussian LPF (depicted in dark green), the normalized strain gradients were successfully retrieved for most of the cases (maximum difference is 20% for aluminium and bovine bone).

In contrast, at SG3 (low-gradient), the average DIC normalized strain gradient and the standard deviation were closer to the reference strain for $\hbox {Stage}_{1}$ and $\hbox {Stage}_{2}$, except for bovine bone at $\hbox {Stage}_{1}$. As well, Gaussian LPF improved the detection of the normalized strain gradients (with maximum difference to the reference of 12%).

Finally, the two experimental strain measurements obtained from DIC and SGs were compared. Table 1 lists the average strain obtained from DIC (at the SG’s position) and the SG’s recorded strain at $\hbox {Stage}_{1}$ and $\hbox {Stage}_{2}$. The average of the standard deviations exceeded 50% for DIC measurements at $\hbox {Stage}_{1}$ where the signal to noise ratio is lowest. One aluminium sample had a very low reading of SG1 at $\hbox {Stage}_{1}$ which resulted in a high standard deviation. A paired-specimens t-test ($\alpha$ = 0.05) was conducted to compare the strain recorded by the SGs and their average corresponding area from DIC. For the majority of the measurements, no significant difference was found between the SGs and the DIC measurements (Statistical summary of the Shapiro-wilk and the t-test can be found in Appendix D, Supplementary Tables 3 and 4). Additionally, the precision of the DIC strain measurement did not change largely and remained between 190 μstrain and 360 μstrain for the different deformation stages.

**Table 1:** Average strain measurements ± standard deviation for the DIC and SGs at the three SGs’ positions. All listed values are in μstrain.

SG	Stage	Aluminium		Polymer		Bovine bone	
		SG	DIC	SG	DIC	SG	DIC
SG1	$\hbox {Stage}_{1}$	217 ± 215	314 ± 193	358 ± 32	260 ± 143	440 ± 115	298 ± 229
SG2		494 ± 7	484 ± 270	497 ± 5	496 ± 172	489 ± 3	544 ± 302
SG3		369 ± 62	459 ± 280	416 ± 54	376 ± 214	472 ± 69	429 ± 203
SG1	$\hbox {Stage}_{2}$	979 ± 398	1055 ± 252	1224 ± 106	1212 ± 236	1329 ± 399	1058 ± 362
SG2		1748 ± 16	1764 ± 224	1743 ± 6	2157 ± 186*	1745 ± 5	1774 ± 276
SG3		1306 ± 147	1503 ± 247	1438 ± 162	1594 ± 227	1775 ± 88	1513 ± 187

The standard deviation for the DIC measurements is the average of the different standard deviations for the measured specimens.

*Indicates a significance difference in the mean.

## Discussion

The main goals of this study were to examine the capability of a 3D DIC system to measure a linear strain field on the surface of a newly designed gradient test specimen, with and without filtering.

Two strain measurement (SGs and DIC) and two strain computational (analytical and FE) methods were employed in this study. The RMSE was evaluated for the high- and low-gradient ROIs at two deformation stages, and the gradient was verified by a newly defined normalized strain gradient measure.

Due to the noise in the DIC strain measurement, the DIC strain deviated ($\hbox {RMSE}_{\text {DIC}}$ < 500 μstrain) from the FE strain - the gold standard in this study - for both the high- and low-gradient ROIs. Gaussian LPF successfully reduced the noise in the DIC full-field strain measurements for all the tested materials. However, the overall reduction in noise can be seen as reduction of the fluctuations of each field rather than reducing the overall measurement values. At $\hbox {Stage}_{1}$ and $\hbox {Stage}_{2}$, the $\hbox {RMSE}_{\text {DIC}}$ was reduced on average by 63% and 34%, respectively. In total, filtering reduced the RMSE to less than 200 μstrain which is in line with values reported in the literature^8,22,32,59^.

The main interest in this work is to examine the capability of DIC to measure strain gradients. For this, two engineering materials, stiffer (aluminium) and softer (polymer) than bone, were chosen for this investigation. At the location of high normalized strain gradient (6.9% per mm), only with applying Gaussian LPF the normalized strain gradient was retrieved. In contrast, at the location of low normalized strain gradient (3.6% per mm), the normalized strain gradient was measured accurately for all cases except for bovine bone, which was then improved when the LPF was applied. The normalized strain gradient is an indicator for how good can the DIC strain measurements measure local variation in the strain fields. In the examined cases, the accuracy of such a value was demonstrated to be higher for low strain concentrations. This can be helpful for measuring strain concentration on the surface of the human femur. However, one should be aware that to decide whether such a value is accurately measured or not, a reference value or an idea of the strain concentration must be known.

Taking a closer look at these analysis, one could look at the DIC full-field strain measurements (see Appendix C, Supplementary Fig. 2). Linear strain fields were not recognized on the surface of all tested specimens. For engineering materials, the deviation from the reference FE was less evident than for the bovine bone specimens, where shifts in the linear strain fields were observed. These shifts can be attributed to the local variations of material and structural properties of bone i.e. the orthotropy of the material or the bone texture. During bone specimen preparation, pores (holes) were visible under the light microscope on the test surface. These holes, originated from blood vessels canals or trabecular bone, influenced the DIC measurements. It might be helpful to reduce the surface roughness by grinding the specimens, however, with grinding, pores might disappear or increase in size and new pores might appear. The non-homogeneity in the measured strain on bone surface was detected by Grassi et al.^51^ who showed that DIC measured strain localization in proximity of cortical pores of the proximal femur. As well as Katz et al.^41^ who showed that holes affected the strain pattern in the DIC measurements and that FE models should consider these holes. This non-homogeneity in the material besides the anisotropic nature of bone contributed to the variations in the DIC strain measurements. It would be useful for future studies to include pores in the FE analysis or create FE models based on geometries obtained from scanned specimens.

There was no significant difference in means between the averaged DIC strain measurement over the SG’s locations and the SGs strain for the majority of the cases. However, no significant difference does not necessarily mean accurate, no clear over- or underestimation were recognized of the DIC measurements. Similar results were found in the literature that validated DIC measurements with SGs or FE models^9,15,22,23^. All these studies suggest that DIC strain measurement should be examined for accuracy. It is obvious that non-consistent errors were found in the DIC measurements, which empathizes the need for validation and optimization to maintain the error at minimum levels.

In summary, this study provided an unprecedented insight into the measurement accuracy of linear strain fields on the surface of different materials by means of DIC. A new innovative specimen shape with two gradients was presented which can be further developed and adapted for different strain gradients and tests with different DIC systems. With the normalized strain gradient, it was possible to measure and verify local strain concentrations which is due to the specimen shape their magnitude were known a priori. This study showed that DIC systems can be optimized for non-homogeneous strain fields such as strains found on the surface of many biological tissues and structures. The normalized strain gradient is essential to understand the range of strain changes per unit mm on the surface of bone. Finally, the common practice of averaging the strain measured on bone surface is not optimal since many strain concentration locations get homogenized.

Limitations of this study are, only one facet and grid size were used as recommended by the DIC software, changing the facet and the grid size to smaller ones would definitely increase the density of the DIC measured points, but at the cost of more noise^11,15,59,60^. Optimal filter parameter found in our previous study^17^ was applied, however, other filter strategies might be useful to reduce the error in DIC strain measurements such as pre-filtering of the speckled images^14^. Finally, the strain gradients values were evaluated for one human proximal femur at physiological load, it might be useful to evaluate strain concatenations beyond the physiological load.

## Conclusion

The normalized strain gradient found on the proximal femur under physiological load was the basis for designing the specimens tested in this work. It was possible to capture such gradients with DIC. Gaussian low-pass filtering reduced the noise found in the DIC measurements and highly improved the detection of the normalized strain gradients. The outcome was better for (1) a lower normalized strain gradient, (2) higher strain level, (3) engineering materials. Beside this finding, the study provides a new specimen design and methodological approach for investigating non-homogeneous full-field strains with DIC on engineering but also hard biological tissues like bone.

## Supplementary Information

Supplementary Information.
