# Supplementary material for: A novel specimen shape for measurement of linear strain fields by means of digital image correlation
Source: Sci Rep. 2021 Sep 1;11:17515. doi: 10.1038/s41598-021-97085-x (PMC8410939; doi:10.1038/s41598-021-97085-x)
Supplement: Supplementary file 1 — Supplementary Information. [file 41598_2021_97085_MOESM1_ESM.pdf]

A novel specimen shape for measurement of linear strain fields by means of digital image correlation

Nedaa Amraish, Andreas Reisinger, Dieter Pahr

SUPPLEMENTARY MATERIAL

A Normalized strain gradient

Supplementary Figure 1 shows a SED map of a proximal femoral under physiological load, the equivalent strains $\varepsilon_{\mathbf{p1}}$ and $\varepsilon_{\mathbf{p2}}$ were computed from SED values at different locations on the surface of the femoral neck and head, two points are shown in Fig. 1 below. The equivalent strain ε_{eq} measured at different locations of **p1** and **p2** on the femur surface and head are listed in Supplementary Table 1, Leading to average normalized strain gradients for the neck and head region of about 7.3 and 3.5% per mm.

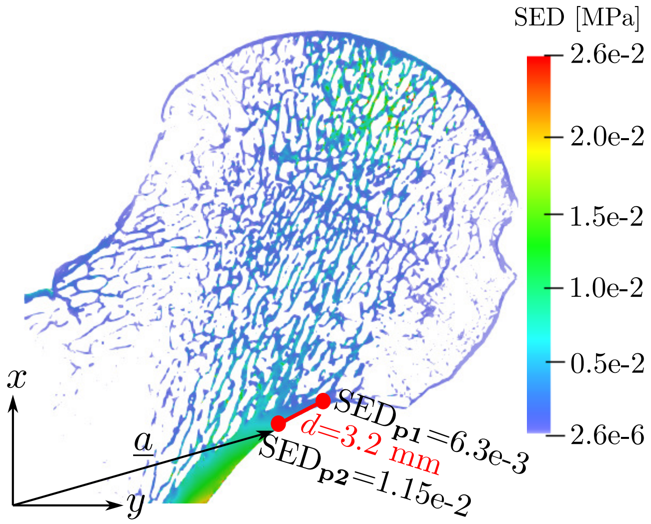

Supplementary Figure 1: Strain energy density map from which the equivalent strain was computed and was employed for the computation of the normalized strain gradient.

Supplementary Table 1: Normalized strain gradient computed from the equivalent strain, all strain values are in μstrain.

$\varepsilon_{\mathbf{p1}}$	$\varepsilon_{\mathbf{p2}}$	$\lvert\varepsilon_{\mathbf{p1}} - \varepsilon_{\mathbf{p2}}\rvert$	$\frac{\varepsilon_{\mathbf{p1}}+\varepsilon_{\mathbf{p2}}}{2}$	**d[mm]**	$[\frac{\%}{\mathrm{mm}}]$
1288	860	428	1074	3.27	12.16
1483	1311	171	1397	3.05	4.03
1655	1378	276	1516.5	3.26	5.60
1342	1039	302	1190.5	3.50	7.26
Average normalized strain gradient - neck region					7.26 $[\frac{\%}{\mathrm{mm}}]$
948	989	41	968.5	3.27	1.30
748	848	100	798	3.05	4.12
860	761	98	810.5	3.26	3.73
1010	848	161	929	3.5	4.96
Average normalized strain gradient - head region					3.53 $[\frac{\%}{\mathrm{mm}}]$

B Elastic modulus

The obtained elastic modulus for each of the tested specimens are listed in Supplementary Table 2. For aluminium and polymer specimens, the measured values are in agreement with the manufacturer. And for bovine bone, results are in agreement with values found in the literature for cortical bone.

Supplementary Table 2: Elastic modulus obtained from SG2 for each of the tested specimens.

**specimen #**	**Aluminium**	**Polymer**	**Bovine bone**
	**Elastic Modulus [GPa]**		
1	68.57	3.12	17.69
2	73.99	3.60	16.44
3	71.02	2.98	17.51
4	70.74	3.10	16.54
5	72.52	3.41	16.45

C Full-field strain measurements

Full-field FE reference strain and DIC (original and filtered) full-field strain measurements are shown in Supplementary Fig. 2.

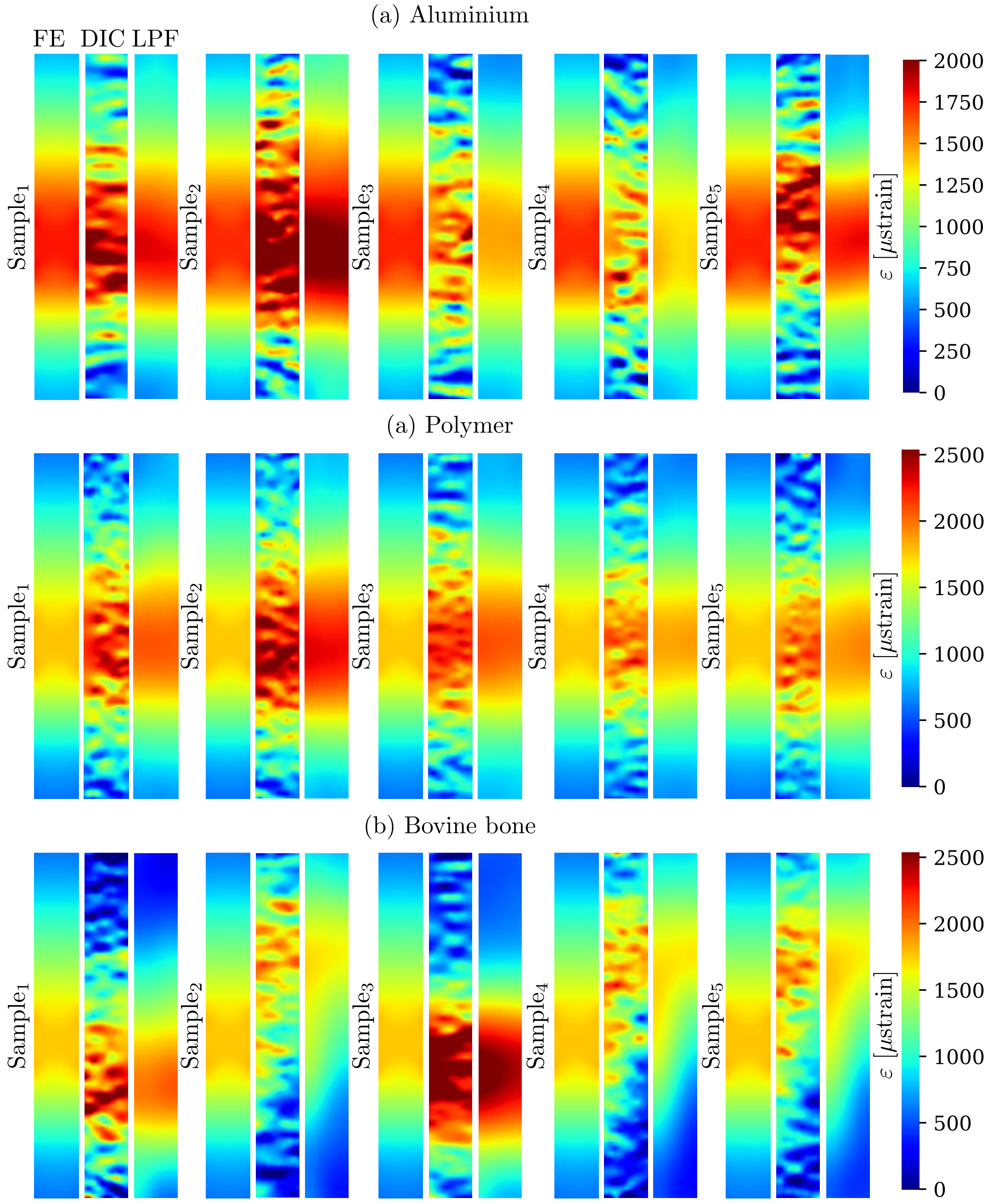

Supplementary Figure 2: 2D visualization of the DIC full-field strain measurements of all tested specimens. At Stage_2; the reference strain from the FE model, the DIC strains (original and filtered) are shown.

D Statistical summary of DIC and SGs' strains

Statistical summary of the normality shapiro-wilk test and paired t-test for Stage_1 and Stage_2 are listed in Supplementary Table 3 and 4, respectively. In the majority of the cases the specimens were normally distributed and no significant difference in the means were found between the DIC averaged strain and the SGs readings.

Supplementary Table 3: Statistical analysis of SGs and DIC data at $Stage_1$. The p value is listed for both the Shapiro-Wilk test and the t-test for normality and different means, respectively.

**Material**	**SG**	**Shapiro-Wilk test**		**t-test**
		**SG**	**DIC**	
Aluminium	SG1	0.392	0.107	0.480
	SG2	0.593	0.971	0.912
	SG3	0.311	0.99	0.307
Polymer	SG1	0.479	0.203	0.116
	SG2	0.842	0.172	0.981
	SG3	0.308	0.324	0.595
Bovine bone	SG1	0.133	0.635	0.448
	SG2	0.982	0.99	0.446
	SG3	0.131	0.970	0.602

Supplementary Table 4: Statistical analysis of SGs and DIC data at $Stage_2$. The p value is listed for both the Shapiro-Wilk test and the t-test for normality and different means, respectively. * indicates a significant difference.

**Material**	**SG**	**Shapiro-Wilk test**		**t-test**
		**SG**	**DIC**	
Aluminium	SG1	0.970	0.148	0.771
	SG2	0.234	0.344	0.925
	SG3	0.278	0.993	0.264
Polymer	SG1	0.084	0.205	0.812
	SG2	0.691	0.621	0.006*
	SG3	0.618	0.987	0.264
Bovine bone	SG1	0.043*	0.467	0.509
	SG2	0.213	0.375	0.906
	SG3	0.967	0.110	0.212
